# The Role of Pleural Fluid C-Reactive Protein in the Diagnosis of Exudative Pleural Effusions

**DOI:** 10.7759/cureus.27000

**Published:** 2022-07-19

**Authors:** Sanket Makwana, Prashant Gohil, Yash Gabhawala

**Affiliations:** 1 General Medicine, C. U. Shah Medical College, Surendranagar, IND; 2 Pulmonary Medicine, C. U. Shah Medical College, Surendranagar, IND

**Keywords:** tuberculous effusion, parapneumonic effusion, exudative effusion, c-reactive protein (crp), pleural effusion

## Abstract

Background and objective

Pleural effusion develops when there is disequilibrium between pleural fluid formation and absorption. Light's criteria are currently used to differentiate transudative from exudative effusion. If the pleural effusion is exudative, it requires extensive diagnostic workup to identify the local cause of the effusion. Pleural fluid cell count and differentials, glucose level, adenosine deaminase (ADA), fluid GeneXpert for Mycobacterium tuberculosis (MTb), fluid culture, and cytology are currently used for further evaluation of exudative pleural effusions. However, the sensitivity and specificity of the above tests are not dependable. The pleural fluid C-reactive protein (CRP) is likely to reflect serum CRP levels because the CRP in the pleural fluid may be caused by increased diffusion from the blood due to inflamed capillary leakage. In this study, we aimed to examine the role of pleural fluid CRP levels in the differential diagnosis of exudative effusion.

Materials and methods

Based on Light's criteria, this study included 100 patients with exudative pleural effusion. Serum CRP and pleural fluid CRP were assessed with the CRP-Turbilatex-quantitative turbidometric immunoassay method based on the principle of an agglutination reaction. Receiver operating characteristic (ROC) curves were generated by plotting sensitivity against 1-specificity, and the area under the curve (AUC) with a 95% confidence interval (CI) was calculated. After data collection, statistical analysis was performed using SPSS Statistics v28.0 (IBM, Armonk, NY).

Results

Our study showed a significant difference in pleural fluid CRP levels (p<0.001). Pleural fluid CRP was significantly higher in the empyema and parapneumonic groups compared to tuberculous and malignant effusions. The optimal cut-off value of CRP ≥47.4 mg/dl yielded 87.5% sensitivity and 92.5% specificity in differentiating parapneumonic effusion from tuberculous effusion. Pleural fluid CRP proved to be an excellent marker for distinguishing parapneumonic effusion from malignancy (cut-off value ≥49.2 mg/dl, 75% sensitivity, and 85.7% specificity) and parapneumonic plus empyema from tuberculous effusion plus malignant effusion (cut-off value ≥47.4 mg/dl, 84.6% sensitivity, and 90.8% specificity).

Conclusion

Pleural fluid CRP levels can be used as an additional tool in the differential diagnosis of exudative effusion. It significantly differentiates parapneumonic effusion and empyema from tuberculous and malignant effusions.

## Introduction

Pleural effusion occurs due to the disequilibrium between pleural fluid formation and absorption [1]. Determining whether the pleural effusion is transudative or exudative is the first step in managing pleural effusion. Exudative pleural effusion should meet at least one of Light's criteria [pleural fluid protein divided by serum protein greater than 0.5, pleural fluid lactate dehydrogenase (LDH) divided by serum LDH greater than 0.6, or pleural fluid LDH greater than two-thirds of the upper limit of normal serum LDH]. In contrast, transudative pleural effusions have to meet none of the criteria [2].

Exudative pleural effusion occurs when local factors influence the formation and absorption of pleural fluid [1]. If the pleural effusion is exudative, it requires extensive diagnostic workup to define the local cause of the effusion [3]. The most common causes of exudative pleural effusion in India are tuberculosis (TB), parapneumonic effusion, malignancy, and empyema [4].

Pleural fluid cell count and differentials, glucose level, adenosine deaminase (ADA), fluid GeneXpert for Mycobacterium tuberculosis (MTb), fluid culture, and cytology are currently employed in the further evaluation of exudative pleural effusions [5,6]. However, the sensitivity and specificity of the above tests are not dependable [7,8]. Pleural fluid cultures provide definitive evidence of parapneumonic effusion and empyema, but their positivity rate is only 60% and they are time-consuming [9,10]. Pleural fluid cytology has a remarkably high false-negative rate [11]. Hence, several novel biomarkers are being studied to establish a cost-effective and rapid method to differentiate between exudative pleural effusions [5,10].

C-reactive protein (CRP) is an acute-phase protein synthesized mainly by hepatocytes in response to various stimuli like bacterial infections, inflammation, malignancy, and pulmonary embolism [12,13]. Measurement of CRP levels is a clinically valuable screening test for organ disease, index of severity, and measure of response to therapy [12]. The pleural fluid CRP is likely to reflect serum CRP levels because the CRP in the pleural fluid may be due to increased diffusion from the blood resulting from inflamed capillary leakage [12,14].

Multiple studies have been conducted regarding the role of pleural fluid CRP in diagnosing exudative pleural effusion worldwide [3,5,10,15]. But in India, where the common causes of exudative effusion differ from those in developed countries, only a few studies are available, with limited samples [4]. In light of this, this study was conducted to identify the efficacy of pleural fluid CRP as a diagnostic biomarker in distinguishing between the etiologies of exudative pleural effusions.

## Materials and methods

Study design and ethical approval

This was a cross-sectional observational study conducted at the C.U. Shah Medical College and Hospital, Surendranagar. Ethical approval was obtained from the Institutional Ethics Committee of the C.U. Shah Medical college with reference no. CUSMC/IEC(HR)/RP/2/2022/FINAL APPROVAL/85/2022.

Inclusion and exclusion criteria

Based on Light's criteria, this study included 100 patients with exudative pleural effusion admitted to the General Medicine and Pulmonary Medicine Department from November 2021 to June 2022. Patients below the age of 12 years were excluded from the study. Written informed consent was taken from all patients before including them in the study.

Methodology

A detailed clinical history, physical examination, chest X-ray P/A view, and chest ultrasound (USG) were performed on all patients. All patients were subjected to routine blood investigations like complete blood count, serum glucose level, serum creatinine, HIV, serum protein and serum LDH level, and sputum for acid-fast bacilli (AFB) and culture examination. Thoracocentesis was done after taking all aseptic precautions, and pleural fluid was examined for appearance, white blood cell (WBC) count, differential cell count, ADA level, sugar level, protein level, LDH level, GeneXpert for MTb, fluid culture, and cytological examination in each patient. Serum CRP and pleural fluid CRP were assessed with the CRP-Turbilatex-quantitative turbidometric immunoassay based on the principle of an agglutination reaction using Siemens Dimension EXL 200 clinical chemistry system manufactured in Brookfield (Siemens Healthcare Diagnostics, Brookfield, CT).

Based on their etiology, exudative effusions were classified into four subtypes: tuberculous effusion, parapneumonic effusion, malignant effusion, and empyema. A diagnosis of tuberculous pleural effusion was made based on pleural fluid ADA level >40 IU and lymphocyte-predominant picture in differential cell count with a clinical history suggestive of TB [4]. Malignant pleural effusion was defined as positive pleural cytological examination or pleural biopsy specimen for malignant cells [5]. A diagnosis of parapneumonic effusion was made in patients with clinical, radiological, or microbiological evidence of pneumonia complicated by pleural effusion [5,15]. Empyema was diagnosed as positive pleural fluid culture and the purulent appearance of pleural fluid with high pleural fluid cell count [5,15].

Statistical analysis

Data were entered into Microsoft excel and categorized and refined as per inclusion and exclusion criteria. Data were presented as means ±standard deviation (SD) for normal distribution and as median with interquartile ranges (IQR) for skewed data. The Kruskal-Wallis test was used for comparisons involving more than two groups. A p-value <0.05 was considered statistically significant. Receiver operating characteristic (ROC) analysis was studied to evaluate the role of pleural fluid CRP in differential diagnoses of exudative pleural effusion. ROC curves were generated by plotting sensitivity against 1-specificity, and the area under the curve (AUC) with a 95% confidence interval (CI) was calculated. After data collection, statistical analysis was performed using SPSS Statistics v28.0 (IBM, Armonk, NY).

## Results

Based on the inclusion and exclusion criteria, 100 patients with exudative effusion were included in the study: 74 males and 26 females. In our research, the most common etiology of exudative effusion was TB (80 patients). Parapneumonic and malignant effusion was found in eight and seven patients, respectively. Empyema was observed in five patients.

This study observed a significant difference in pleural fluid WBC count, which was highest in the empyema group, followed by the parapneumonic group. The pleural fluid WBC count was lowest in malignant effusion. There was also a significant difference in the groups' pleural fluid lymphocytes and neutrophils. The parapneumonic and empyema groups had a higher pleural fluid neutrophil percentage, while tuberculous and malignant effusion had higher pleural fluid lymphocyte percentages (Table 1).

The biochemical analysis of pleural fluid showed a significant difference in pleural fluid protein, pleural fluid LDH, and pleural fluid ADA levels among the groups. The ratio of pleural fluid protein to serum protein was highest (1.26) in the empyema group. The pleural fluid LDH to serum LDH ratio was also the highest (30.45) in the empyema group, followed by parapneumonic effusions (4.19). Pleural fluid ADA was significantly higher in tuberculous effusion, followed by empyema (Table 1).

**Table 1:** Demographic data and pleural fluid characteristics Data are presented as mean ±SD for normally distributed data or median (interquartile range) for skewed data. P-value <0.05 considered statistically significant WBC: white blood cell; LDH: lactate dehydrogenase; ADA: adenosine deaminase; SD: standard deviation

Characteristics	Tuberculous effusion	Parapneumonic effusion	Malignant effusion	Empyema	P-value
Number (n)	80	8	7	5	~
Male, n (%)	58 (72.5%)	5 (62.5%)	6 (85.7%)	4 (80%)	~
Age (years)	45.55 ±14.11	36.5 (35.5-52)	70 ±8.35	48 (47-52)	0.00132
Amount (ml)	900 (550-1400)	1650 (1450-1800)	1100 (850-1400)	1500 (1300-1500)	0.00156
Pleural fluid WBC (×10^6^/L)	3150 (1150-5650)	4700 (1800-6650)	600 (250-5800)	24500 (14300-29125)	0.00556
Pleural fluid neutrophils (%)	8 (5-33)	88.5 (82-90.5)	7 (1-9)	87 (82.5-87.5)	<0.00001
Pleural fluid lymphocytes (%)	85 (62-91)	8 (6.5-14)	70 (68-88)	13 (11.5-14)	0.00001
Pleural fluid glucose (mg/dl)	78 (68.5-92)	79.63 ±6.44	88 (68-129)	68 (66-76.5)	0.29415
Pleural fluid protein (g/dl)	5.2 (4.47-5.6)	3.94 (3.49-4.1)	4.28 ±0.89	7.8 (5.46-7.88)	0.00029
Serum protein (g/dl)	5.59 (5.24-5.92)	5.01 ±0.44	4.8 (4.56-5.23)	6.2 (5.41-6.27)	0.00051
Pleural protein/serum protein ratio	0.92 (0.84-1.02)	0.80 (0.71-0.82)	0.85 (0.71-0.93)	1.26 (0.90-1.39)	0.00769
Pleural fluid LDH (U/L)	392 (293.8-606.5)	858.4 (754.5-3015.2)	419 (310.2-480.2)	8500 (7582-9122.5)	0.00005
Serum LDH (U/L)	213.5 (177.2-287.5)	252 (139.2-397.6)	345 (262-422)	311 (211-314.5)	0.01891
Pleural LDH/serum LDH ratio	1.74 (1.19-3.02)	4.19 (2.36-13.74)	1.30 (0.87-1.65)	30.45 (27.46-35.93)	0.00013
Pleural fluid ADA (U/L)	87.1 (68.1-121.15)	54.46 ±16.56	27.11 ±1.78	58.22 ±13.9	<0.00001

Our study showed a significant difference in pleural fluid CRP among the groups. Pleural fluid CRP was significantly higher in the empyema and parapneumonic groups compared to tuberculous and malignant effusions. However, there was no significant difference in pleural fluid CRP between the empyema and parapneumonic groups, as well as malignant and tuberculous effusions. The ratio of pleural CRP to serum CRP was non-significant among the exudative groups (Table 2).

**Table 2:** Levels of CRP in pleural fluid and blood Data are presented as mean ±SD for normally distributed data or median (interquartile range) for skewed data. P-value <0.05 considered statistically significant CRP: C-reactive protein; SD: standard deviation

Characteristics	Tuberculous effusion	Parapneumonic effusion	Malignant effusion	Empyema	P-value
Pleural CRP (mg/L)	32.1 (26.25-40.35)	72.36 ±23.25	26.9 (22.1-48.9)	72.96 ±28.36	0.00002
Serum CRP (mg/L)	18.92 (12.46-29.7)	59.05 (53.6-68.15)	19.3 (16.44-24.2)	56.51 ±10.49	<0.00001
Pleural CRP/serum CRP ratio	1.57 (1.13-2.21)	1.06 (0.92-1.44)	1.12 (0.73-2.97)	1.31 (0.88-1.67)	0.15869

We used the ROC curve to determine the diagnostic performance of pleural fluid CRP in the differential diagnosis of exudative pleural effusions. ROC curves were generated by plotting sensitivity against 1-specificity. The AUC with a 95% CI was calculated.

The optimal cut-off value of CRP ≥47.4 mg/dl yielded 87.5% sensitivity and 92.5% specificity in differentiating parapneumonic effusion from tuberculous effusion. Pleural fluid CRP proved to be an excellent marker for distinguishing parapneumonic effusion from malignancy (cut-off value ≥49.2 mg/dl, 75% sensitivity, and 85.7% specificity) and parapneumonic plus empyema from tuberculous effusion plus malignant effusion (cut-off value ≥47.4 mg/dl, 84.6% sensitivity, and 90.8% specificity). However, pleural fluid CRP showed poor diagnostic efficacy in distinguishing malignant effusion from tuberculous effusion (cut-off value ≥34.5 mg/dl, 42.9% sensitivity, and 56.2% specificity) (Table 3).

**Table 3:** Diagnostic performance of pleural fluid CRP based on the ROC analysis CRP: C-reactive protein; ROC: receiver operating characteristic; AUC: area under the curve

Biomarkers	Optimal cut-off value of CRP (mg/dL)	Sensitivity (%)	Specificity (%)	AUC
Parapneumonic vs. tuberculous effusion	≥47.4	87.5	92.5	0.950
Parapneumonic vs. malignant effusion	≥49.2	75.0	85.7	0.902
Parapneumonic effusion plus empyema vs. tuberculous plus malignant effusion	≥47.4	84.6	90.8	0.928
Malignant vs. tuberculous effusion	≥34.5	42.9	56.2	0.443

**Figure 1:**
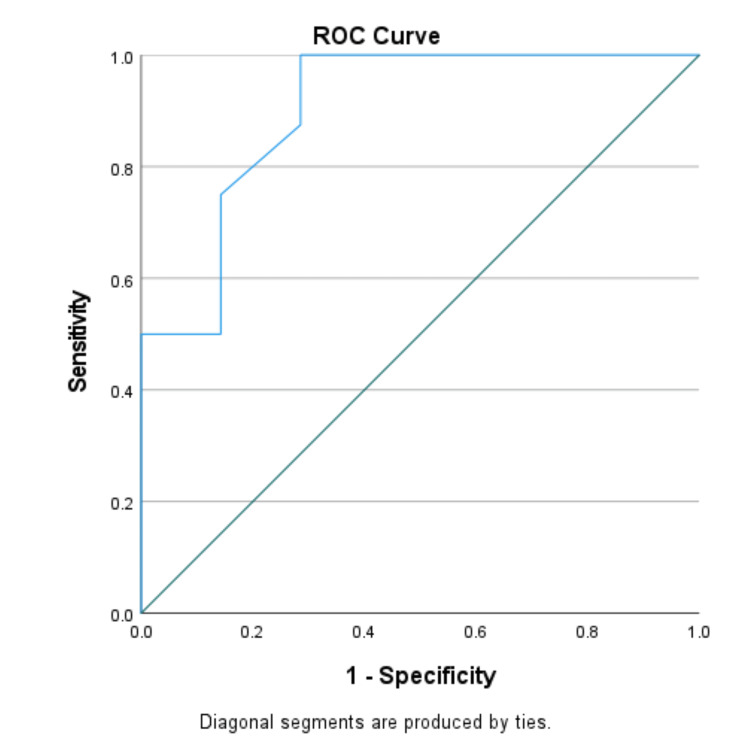
Parapneumonic effusion vs. malignant effusion (AUC: 0.902) ROC: receiver operating characteristic; AUC: area under the curve

**Figure 2:**
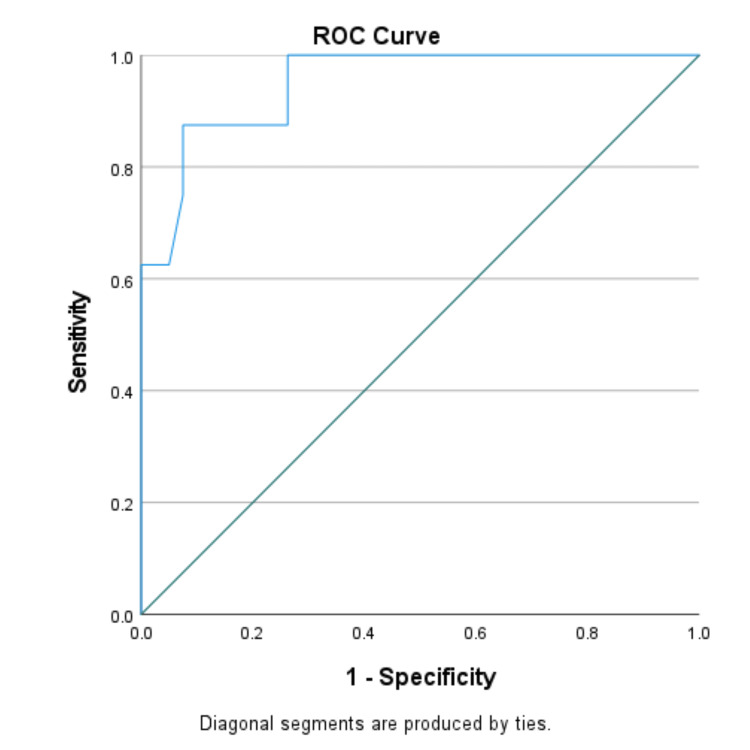
Parapneumonic effusion vs. tuberculous effusion (AUC: 0.950) ROC: receiver operating characteristic; AUC: area under the curve

**Figure 3:**
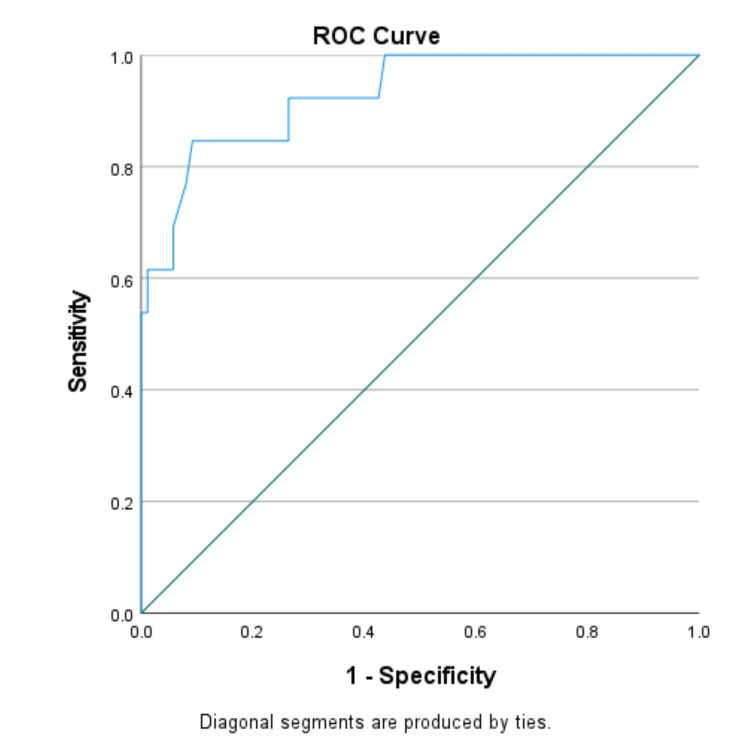
Parapneumonic effusion plus empyema vs. tuberculous plus malignant effusion (AUC: 0.928) ROC: receiver operating characteristic; AUC: area under the curve

**Figure 4:**
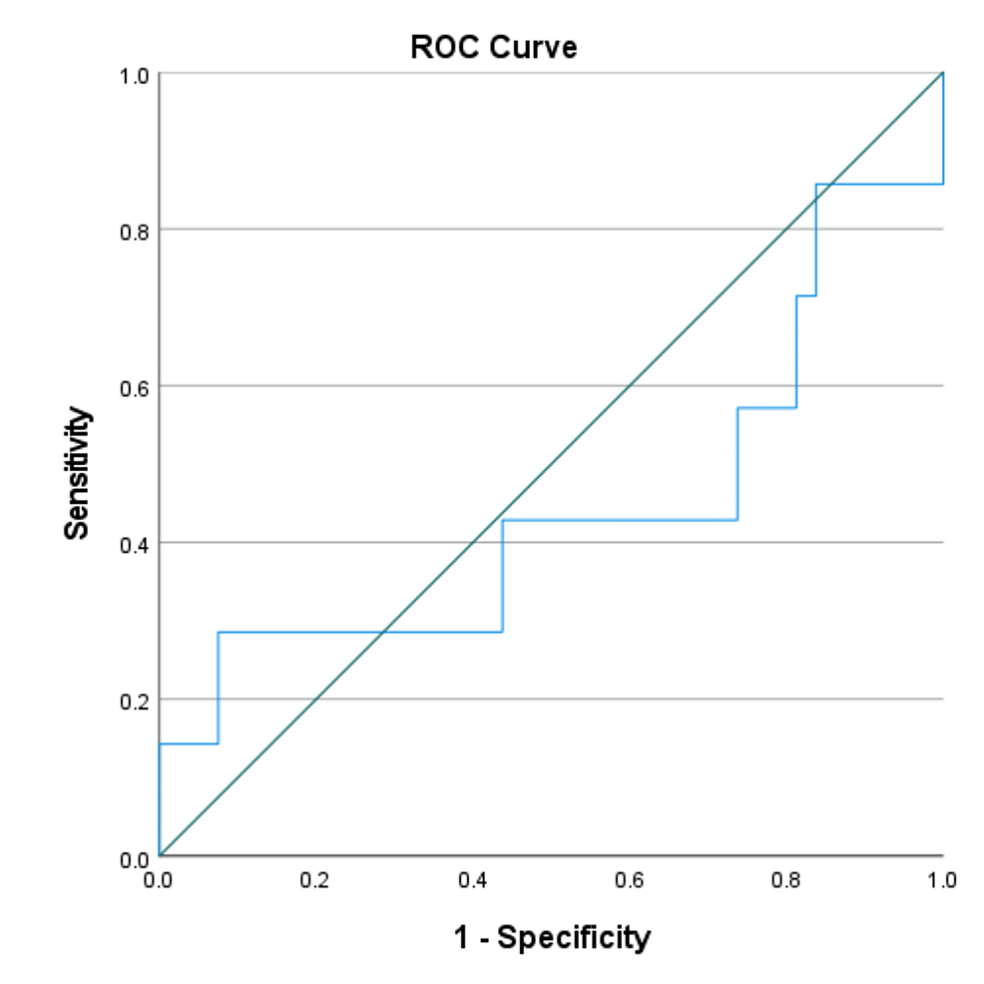
Malignant effusion vs. tuberculous effusion (AUC: 0.443) ROC: receiver operating characteristic; AUC: area under the curve

The AUC for parapneumonic effusion vs. tuberculous effusion, parapneumonic effusion vs. malignant effusion, and parapneumonic effusion plus empyema vs. tuberculous effusion plus malignant effusion were 0.950, 0.902, and 0.928 respectively (Figures 1-3). AUC for malignant vs. tuberculous effusion was only 0.443, showing that pleural fluid CRP has no role in differentiating between malignant and tuberculous effusions (Figure 4).

## Discussion

This study included a total of 100 patients with exudative effusion. Light's criteria are currently used to differentiate transudative from exudative effusion [2]. Exudative pleural effusion is clinically common in various respiratory disorders, and its further sub-classification mainly relies on pleural fluid routine and biochemical, cytological, and pathological examinations [16]. However, the sensitivity and specificity of the above tests are unsatisfactory for achieving a specific diagnosis of exudative effusion. Invasive procedures like pleural biopsy and thoracoscopy give excellent results in identifying the exact etiology of exudative effusion. Still, most patients are unwilling to do it due to its invasive nature. Procedures-related complications are also common with these invasive procedures. Cytological examinations are time-consuming and are associated with high false-negative rates. Hence, there is a need for a novel biomarker that gives a rapid diagnosis with high sensitivity and specificity.

CRP is an acute-phase reactant secreted by hepatocytes and is an important diagnostic test for laboratory screening of infectious and non-infectious diseases. In the present study, we evaluated the role of pleural fluid CRP in the differential diagnosis of exudative effusions.

In our study, patients were in the age range of 20-79 years, with males predominantly affected compared to females. The study by Qu et al. [16] involving 87 patients with exudative pleural effusion also reported that males were more affected (62 cases) than females (25 patients). In our study, the most common cause of exudative effusion was TB (80%), followed by parapneumonic and malignant effusion in 8% and 7% of patients, respectively. Empyema was the least common cause (5%) in our study group. A study conducted by Antonangelo et al. [17] among 326 patients with pleural effusion also reported TB as the most common cause of exudative effusion (55.8%, 126 patients).

Our study has shown a statistically significant difference in pleural fluid protein, pleural fluid LDH, and pleural fluid cell count among the exudative groups. Pleural fluid neutrophils were predominantly observed in the empyema and parapneumonic groups, while pleural fluid lymphocytes were predominant in tuberculous and malignant effusion. These findings are comparable with the results obtained by Watanabe et al. [5] and Qu et al. [16]. Pleural fluid ADA level was significantly higher in tuberculous effusion [87.1 (68.1-121.15)] than in other causes of exudative effusion. Studies by Qu et al. [16] and Radhakrishnan et al. [4] also reported that the pleural fluid ADA level was significantly higher in the tuberculous effusion group.

We found that pleural fluid and serum CRP levels were higher in the parapneumonic and empyema groups compared to tuberculous and malignant effusions. These findings were statistically significant. But our study did not demonstrate a significant difference in terms of pleural CRP/serum CRP ratio (p=0.15869).

To evaluate the diagnostic performance of pleural fluid CRP, we created an ROC curve between sensitivity vs. 1-specificity. To distinguish between parapneumonic and tuberculous effusion, an optimal cut-off value of pleural CRP ≥47.4 mg/dl has a very high sensitivity of 87.5% and highest specificity of 92.5%, with an AUC of 0.950. Pleural fluid CRP at an optimal value of ≥47.4 mg/dl with 84.6% sensitivity and 90.8% specificity was an excellent biomarker in differentiating parapneumonic plus empyema from tuberculous effusion plus malignant effusion. To demarcate parapneumonic effusion from malignancy, we observed 75% sensitivity and 85.7% specificity at a cut-off value of ≥49.2 mg/dl. Our study's ROC analysis showed a poor performance in differentiating malignancy from tuberculous effusion (AUC: 0.443).

Izhakian et al. [15] have reported that parapneumonic effusion had a higher pleural fluid CRP level than other exudative effusions at a cut-off value of >1.38 mg/dl. Porcel et al. [10] also reported that the level of pleural fluid CRP >10 mg/dl was significantly associated with complicated parapneumonic effusions, and they required pleural fluid drainage. In the present study, our cut-off value of parapneumonic effusion from TB was ≥47.4 mg/dl, while a survey by Radhakrishnan et al. [4] used a cut-off value of pleural fluid CRP >70 mg/dl. A study by Gabhale et al. [18] on the usefulness of pleural fluid CRP level in the differential diagnosis of exudative pleural effusion had a pleural fluid CRP cut-off value ≥90 mg/dl for parapneumonic effusion. Various other studies have highlighted the relationship between pleural fluid CRP and the etiology of pleural effusion with similar findings [3,19].

However, the exact cut-off value for pleural fluid CRP is variable among the different studies; pleural fluid CRP level appears to be a potential marker in differentiating the exudative effusion.

This study should be interpreted in the context of certain limitations. In our study group, most of the patients were in the tuberculous groups compared to other groups due to the higher prevalence of TB in our geographical region. Hence, a future multi-centric longitudinal study with a large sample size with a significant number of patients with parapneumonic effusion, empyema, and malignant effusion is required to provide a more powerful impact in terms of distinguishing the etiology of exudative pleural effusions.

## Conclusions

Pleural fluid CRP levels can be used as an additional tool in the differential diagnosis of exudative effusion. It significantly differentiates parapneumonic effusion and empyema from tuberculous and malignant effusions. But the role of pleural fluid CRP in distinguishing malignant effusion from tuberculous effusion was not significant in our study. It is evident from our study that pleural fluid CRP is a very rapid and cost-effective tool to differentiate parapneumonic effusion and empyema from other exudative effusions.
